# Fulminant Lyme Carditis With Rapid Hemodynamic Deterioration in an Adolescent: A Case Report

**DOI:** 10.7759/cureus.104434

**Published:** 2026-02-28

**Authors:** Mohamed Enaimi, Anas Elbouti, Hicham Hammadi, Sara Babour, Noureddine Kartite, Abdelhafid Houba, Bakkali Hicham, Nawfal Doghmi

**Affiliations:** 1 Anesthesiology and Intensive Care, Mohamed V Military Hospital, Rabat, MAR; 2 Pediatric Rheumatology, Mohamed V Military Hospital, Rabat, MAR

**Keywords:** borreliosis, cardiogenic shock, fulminant, lyme carditis, myocarditis

## Abstract

Lyme carditis is a rare but potentially severe complication of Lyme disease caused by *Borrelia burgdorferi*. We report the case of a 16-year-old adolescent with no significant past medical history, admitted for hemodynamic instability in a febrile context associated with respiratory and gastrointestinal symptoms evolving over several days. Transthoracic echocardiography showed marked biventricular dilation with global hypokinesia and a left ventricular ejection fraction estimated at 20-25%, associated with a small pericardial effusion. Laboratory investigations revealed a significant inflammatory syndrome and progressively rising troponin levels.

Appropriate antibiotic therapy combined with inotropic and vasopressor support was initiated after serological confirmation of Lyme disease. Despite initial hemodynamic improvement, secondary deterioration occurred with the development of refractory cardiogenic shock. The clinical course was rapidly unfavorable within less than 12 hours, ultimately leading to death.

This case illustrates the potential severity and fulminant evolution of Lyme carditis, highlighting the importance of early diagnosis, close cardiac monitoring, and timely access to appropriate supportive therapies.

## Introduction

Lyme disease is the most common vector-borne zoonosis in the Northern Hemisphere and is caused by *Borrelia burgdorferi*. Cardiac involvement occurs in a minority of cases and most commonly presents as atrioventricular conduction disturbances that are generally reversible with appropriate antibiotic therapy [1,2].

Although uncommon, Lyme carditis may rarely manifest as acute myocarditis with severe ventricular dysfunction. In exceptional cases, rapid progression to fulminant myocarditis complicated by cardiogenic shock has been described, reflecting both direct myocardial tropism of the organism and associated inflammatory mechanisms [3].

Because initial manifestations are often nonspecific, diagnosis may be delayed, particularly in children and adolescents. These severe presentations remain insufficiently characterized and may deteriorate rapidly despite guideline-based management [4].

We report a fatal case of fulminant Lyme myocarditis in a previously healthy adolescent, characterized by initial transient improvement followed by abrupt hemodynamic collapse. This observation highlights the unpredictable course and potential severity of Lyme carditis, even in the setting of specialized medical care.

## Case presentation

A 16-year-old adolescent with no significant past medical history was admitted to the intensive care unit for the management of hemodynamic instability. The clinical history began with a productive cough, epigastric pain, vomiting, and marked asthenia in a context of intermittent fever. His general condition progressively deteriorated, with the onset of exertional dyspnea that rapidly worsened, followed by several episodes of hemoptysis, prompting an initial evaluation in the pediatric department. Due to persistent symptoms and hemodynamic deterioration, the patient was transferred to the intensive care unit.

**Figure 1:**
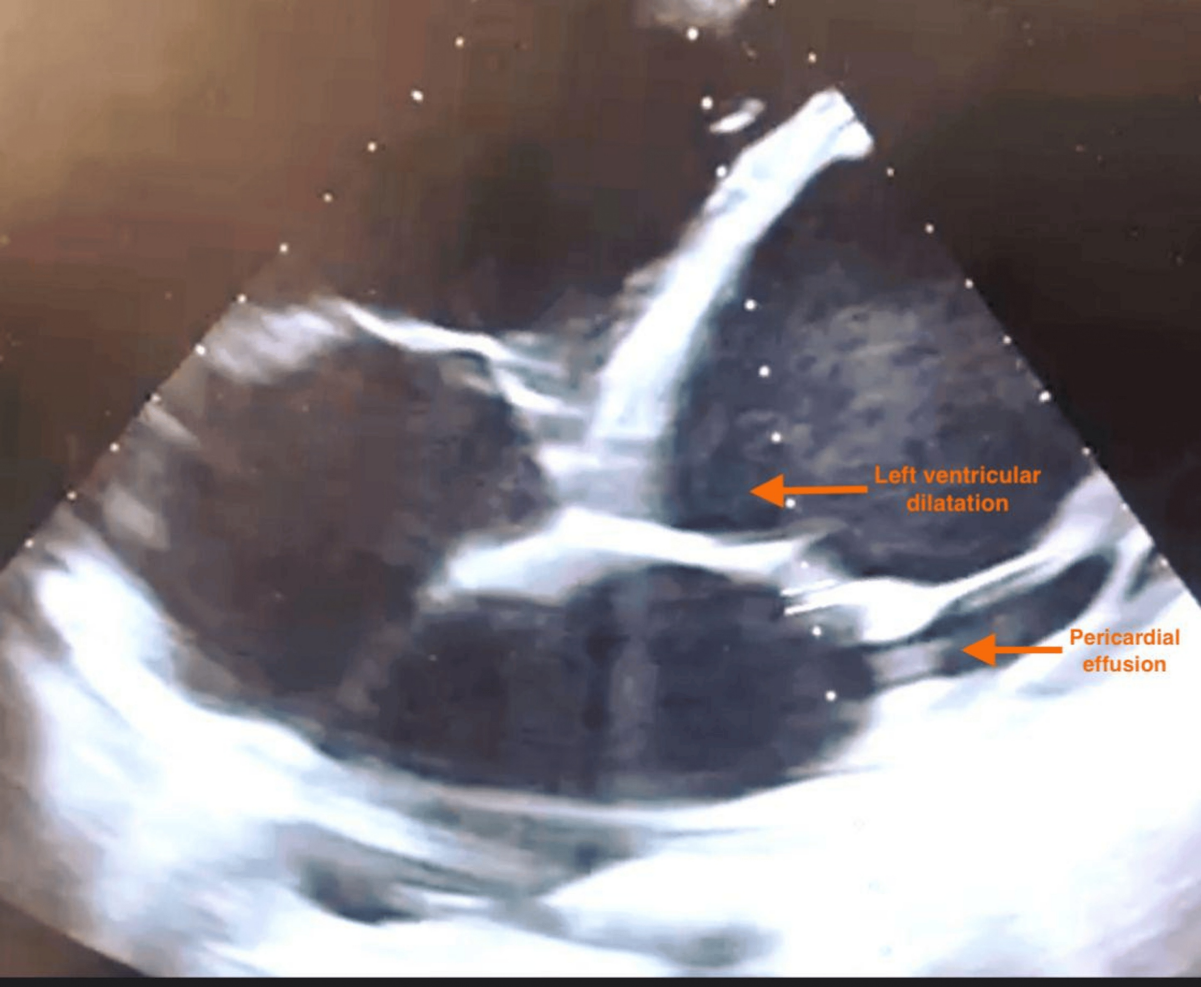
Apical four-chamber transthoracic echocardiographic view demonstrating marked dilatation of the left ventricle and a circumferential pericardial effusion

On admission, he presented with hypotension (blood pressure 84/40 mmHg), sinus tachycardia at 158 bpm, tachypnea at 35 breaths/min, and oxygen saturation of approximately 88-90% under supplemental oxygen. He was conscious but severely dyspneic, with initially preserved capillary refill time. Electrocardiography revealed a rapid sinus tachycardia. Transthoracic echocardiography demonstrated severe left ventricular systolic dysfunction with marked left ventricular dilatation and global hypokinesia, and an estimated left ventricular ejection fraction of 20-25%. The right ventricle was also dilated with impaired systolic function, the tricuspid valve showed mild structural changes, and a small pericardial effusion was present (Figure 1).

Initial laboratory investigations revealed a significant inflammatory syndrome (C-reactive protein: 68 mg/L, white blood cell count: 19,000/mm³), elevated and rising troponin levels, moderately increased lactate levels, and functional acute kidney injury with elevated serum creatinine. The patient also exhibited hyponatremia, severe hypokalemia, and moderate hepatic cytolysis. Serial chest radiographs showed cardiomegaly with bilateral basal alveolar-interstitial opacities.

The initial course was marked by cardiogenic shock requiring urgent initiation of norepinephrine and dobutamine to maintain adequate perfusion pressure. Strict fluid restriction and diuretic therapy with furosemide were implemented. Empirical antibiotic therapy with third-generation cephalosporins and doxycycline was started. Microbiological investigations subsequently returned positive for Lyme disease, confirming the diagnosis of severe Lyme carditis.

Under inotropic and vasopressor support and multidisciplinary management, the patient’s hemodynamic status gradually improved over the first five days. Troponin levels decreased, renal function normalized, and vasoactive drugs were progressively weaned. The patient was then transferred to the pediatric ward for continued management and close monitoring.

However, one week after discharge from the intensive care unit, the patient was readmitted in profound cardiogenic shock. Echocardiography revealed fulminant myocarditis with severe biventricular failure, a more significant pericardial effusion, and a rapid decline in cardiac output. Despite immediate reintroduction of catecholamines and inotropic agents, ventilatory support, and optimized antibiotic therapy, the clinical course was rapidly unfavorable. Within less than 12 hours, the patient developed refractory hemodynamic collapse unresponsive to conventional therapies. Death occurred in the context of irreversible cardiogenic shock secondary to fulminant myocarditis related to Lyme disease.

## Discussion

Lyme carditis is a potentially severe complication of *Borrelia burgdorferi *infection. This case illustrates the fulminant and rapidly progressive form of myocardial involvement that may occur in adolescents, leading to refractory cardiogenic shock despite early antibiotic therapy. Understanding the underlying inflammatory and immune-mediated mechanisms is essential to explain such abrupt deterioration [1].

Increasing evidence suggests that pathophysiology is not solely related to direct bacterial invasion. A significant component of myocardial dysfunction may be mediated by an exaggerated host immune response. Some patients experience delayed clinical worsening despite appropriate antibiotic therapy, supporting the hypothesis of immune-mediated myocardial injury or delayed hypersensitivity reactions [2,3]. This immune-mediated mechanism likely explains the biphasic clinical course reported in several cases, initial transient stabilization followed by sudden hemodynamic collapse, which closely mirrors the evolution observed in our patient.

Cardiac manifestations of Lyme disease are most frequently dominated by atrioventricular conduction disturbances [4,5]. However, myocarditis and pancarditis, although rare, represent the most severe clinical expressions of the disease [2]. Cardiac involvement may be uni- or biventricular, with biventricular failure--present in our patient--being a major prognostic marker of severity. Extreme chamber dilation, profound systolic dysfunction, and the presence of pericardial effusion are all associated with a high risk of progression to cardiogenic shock [3].

In children and adolescents, presentations are often atypical. Fever, cough, abdominal pain, marked asthenia, or gastrointestinal symptoms frequently precede overt cardiac failure. This clinical variability, combined with the frequent absence of erythema migrans (present in fewer than one-third of cases of Lyme carditis [4]), contributes to diagnostic delay, a critical factor in the development of fulminant forms. In our case, the initial respiratory and digestive symptoms oriented the diagnostic approach toward a non-cardiac infectious process, potentially delaying suspicion of Lyme disease.

Diagnosis relies on the integration of epidemiological, clinical, serological, and echocardiographic findings. Echocardiography is the cornerstone investigation, allowing the assessment of ventricular function, chamber dilation, filling pressures, and detection of pericardial effusion. In fulminant forms, left ventricular ejection fraction may deteriorate dramatically within hours, as observed in our patient. Troponin levels are often elevated but nonspecific and correlate more closely with the degree of myocardial inflammation than with bacterial burden [3]. Serological tests confirm the diagnosis but may be negative early in the disease course, emphasizing the need to initiate antibiotic therapy promptly when clinical suspicion is high.

Standard treatment is based on appropriate antibiotic therapy, particularly third-generation cephalosporins or doxycycline, which generally result in favorable outcomes in uncomplicated cases [6]. However, in fulminant forms, antibiotics alone are insufficient. Hemodynamic management must be aggressive and early, including vasopressors, inotropes, mechanical ventilation, and strict fluid management. The literature indicates that, despite optimal medical therapy, fulminant myocarditis may progress to refractory cardiogenic shock in the absence of mechanical circulatory support [7].

Veno-arterial extracorporeal membrane oxygenation (VA-ECMO) is currently considered the cornerstone of treatment for pediatric fulminant myocarditis and severe Lyme carditis. It allows complete myocardial rest, immediate improvement in cardiac output, and maintenance of systemic perfusion during the acute inflammatory phase [7]. Several case reports have described full recovery of ventricular function after 5-10 days of support [8], confirming the potentially reversible nature of myocardial injury when support is initiated in a timely manner. In our case, clinical deterioration was exceptionally rapid, with fulminant hemodynamic collapse occurring within less than 12 hours, precluding timely initiation of mechanical circulatory support despite clear indication. The principal clinical lesson from this case is that Lyme carditis can deteriorate catastrophically within hours, even after apparent initial stabilization.

Finally, this case emphasizes the need to systematically consider Lyme disease in the differential diagnosis of acute febrile myocarditis, particularly in endemic areas. It also serves as a reminder that Lyme disease, often perceived as a benign condition, can rarely progress to life-threatening cardiac complications. Early recognition, prompt initiation of appropriate antibiotic therapy, and timely access to mechanical circulatory support are key elements in improving patient outcomes.

## Conclusions

This case reports a fulminant form of Lyme carditis in an adolescent, characterized by rapid hemodynamic deterioration leading to refractory cardiogenic shock. Despite early initiation of appropriate antibiotic therapy and intensive hemodynamic support, the clinical course worsened within hours, culminating in non-recoverable cardiac arrest. Transthoracic echocardiography demonstrated severe left ventricular dilatation associated with a small pericardial effusion, consistent with acute inflammatory myocardial involvement. This observation highlights the potential for abrupt and catastrophic progression of Lyme carditis, even in previously healthy young patients, and underscores the importance of early recognition, close monitoring, and timely consideration of advanced mechanical circulatory support in fulminant presentations.
